# Comprehensive analyses of fatty acid metabolism-related lncRNA for ovarian cancer patients

**DOI:** 10.1038/s41598-023-35218-0

**Published:** 2023-09-06

**Authors:** Min Li, Ye Yan, Yanyan Liu, Jianzhen Zhao, Fei Guo, Jianqin Chen, Lifang Nie, Yong Zhang, Yingmei Wang

**Affiliations:** 1 Department of Gynecology and Obstetrics, Tianjin Medical University General Hospital, 154 Anshan Road, Heping, Tianjin, 300052 China; 2 Tianjin Key Laboratory of Female Reproductive Health and Eugenics, Tianjin Medical University General Hospital, Tianjin, 300052 China; 3 Department of Gynecology, Jincheng People’s Hospital, Jincheng, 048026 China; 4 Department of Pathology, Jincheng People’s Hospital, Jincheng, 048026 China

**Keywords:** Cancer, Molecular biology

## Abstract

Ovarian cancer (OC) is a disease with difficult early diagnosis and treatment and poor prognosis. OC data profiles were downloaded from The Cancer Genome Atlas. Eight key fatty acid metabolism-related long non-coding RNAs (lncRNAs) were finally screened for building a risk scoring model by univariate/ multifactor and least absolute shrinkage and selection operator (LASSO) Cox regression. To make this risk scoring model more applicable to clinical work, we established a nomogram containing the clinical characteristics of OC patients after confirming that the model has good reliability and validity and the ability to distinguish patient prognosis. To further explore how these key lncRNAs are involved in OC progression, we explored their relationship with LUAD immune signatures and tumor drug resistance. The structure shows that the risk scoring model established based on these 8 fatty acid metabolism-related lncRNAs has good reliability and validity and can better predict the prognosis of patients with different risks of OC, and LINC00861in these key RNAs may be a hub gene that affects the progression of OC and closely related to the sensitivity of current OC chemotherapy drugs. In addition, combined with immune signature analysis, we found that patients in the high-risk group are in a state of immunosuppression, and Tfh cells may play an important role in it. We innovatively established a prognostic prediction model with excellent reliability and validity from the perspective of OC fatty acid metabolism reprogramming and lncRNA regulation and found new molecular/cellular targets for future OC treatment.

## Introduction

Ovarian cancer is one of the most familiar gynecological cancers and has the highest death rate, killing about 150,000 women every year^1,2^. Due to short of representative clinic features as well as early predictive biomarkers, almost seventy percent of OC patients with advanced stage were diagnosed and had a bad prognosis^3^. More than 70% patients experience recurrence after treatment as well as five years’ living rate of OC is lower than 30%^4^. Therefore, it is necessary to identify accurate molecular biomarkers to improve the prognosis and treatment sensitivity of OC patients.

The long non-coding RNAs (lncRNAs) have transcripts of over two hundred nucleotides, which can be shown to exert important roles in tumorigenesis as well as development^5–7^. Recently, more and more researches have shown lncRNAs may be related to OC processes^8–10^. Such as, lncRNA LINC00504 facilitates OC cell development and motivates aerobic glycolysis through coaction with miR-1244^11^. LncRNASNHG12 and HOTTIP were found to assist ovarian cancer cells with escaping the immune system^12,13^. Additionally, lncRNAs are emerging as potential biomarkers of OC, promoting drug resistance, relapse as well as result in bad prognosis^14^. Zheng et al. constructed a N6-methyladenosine-related lncRNA model to improve the predictive value for patients with OC^15^. Similarly, Zhao et al. also found 5 lncRNAs that were highly associated with OC^16^. The newly identified signatures related to lncRNAs have provided guidance for prognosis prediction and enhanced clinical treatment outcomes.

Due to the imbalance between rapid proliferation of tumor cells and nutrient angiogenesis, tumor cells often thrive in an abnormal metabolic environment^17^. Metabolic reprogramming of tumor cells is an important marker, and glutamine metabolism, changes in aerobic glycolysis (Warburg effect), oxidative phosphorylation and 1 carbon pathway also enhance the ability of tumor to progress rapidly in tumor microenvironment under relative nutritional stress^18–21^. Additionally, increasing attention has been paid to abnormal fatty acid metabolism as a typical metabolic reprogram of cancer^22,23^. For example, Zhao et al. found that SIK2 promotes fatty acid synthesis in OC cells via PI3K/Akt signal pathway^24^. However, the regulation of lncRNA on fatty acid metabolism pathways in OC is unknown. Further understanding the function of aliphatic acid metabolism related lncRNAs of OC might provide a deep insight into potential mechanisms and find accurate treatment.

In this study, OC samples and patient-related information could be downloaded from TCGA database. Finally, 8 lncRNAs are selected, and a risk scoring model is established by uni-factor/multi-factor least Absolute and Selection Operator (LASSO) Cox regression. The nomogram could be established for next clinical usage. To further explore how these key lncRNAs are involved in OC progression, we explored their relationship with OC immune signatures and tumor drug resistance. Function enriching study was also performed to searching for underlying OC progression mechanisms.

## Methods and materials

### Data acquisition

LncRNA and mRNA expression as well as clinic data from OC patients could be downloaded from TCGA database (https://portal.gdc.cancer.gov/) ^25^. To reduce statistical bias, OC patients’ samples of lost overall survival (OS) or short OS (< 30 days) were eliminated. Then we obtained 364 samples and were divided in training (n = 184) as well as testing sets in random (n = 180), and the two datasets are consistent in clinical characteristics (*P* > 0.05). Training part was applied to build prognostic model as well as testing part was applied to validate model. Additionally, all 92 fatty acid metabolism related genes could be obtained from Gene Cards (https://www.genecards.org) with a relevance score ≥ 7. A fatty acid metabolism-related lncRNA was identified with Pearson’s correlation analysis following the criterion of |Pearson R|> 0.3 and *P* < 0.001. The correlation between them was visualized using the 'heatmap' package of R (version 4.1.1). All data were obtained from open-access databases, thus no medical ethics committee approval was required.

### Construction and validation of the risk signature

Univariate Cox regression could be performed for screening lncRNAs associated with fatty acid metabolism-relating lncRNA. Next, LASSO regression was performed to screen consequences of univariate Cox regression by the R package ‘glmnet’^26,27^. Eleven fatty acid metabolism-relating lncRNAs cleared associated with OS in OC patients from TCGA datasets were identified by LASSO regression. Multifactor Cox regression was used to study 11 fatty acid metabolism-related metabolism-relating lncRNAs, and 8 fatty acid metabolism-related risk model was ultimately established.

We counted risking scores in risk model by using formula as followings:

$$ Risk score = \mathop \sum \limits_{k = 1}^{n} Coef\left( {lncRNA} \right)*expr\left( {lncRNA^{k} } \right) $$

where the coef (lncRNA) was the short form of the coefficient of lncRNAs correlated with survival and expr (lncRNAn) was the expression of lncRNAs. Based on the median risk score, subgroups including low- and high-risk groups were established. Where, Coef (lncRNA) was the short form of lncRNAs and survival correlation coefficient, expr (lncRNAn) was the expression of lncRNAs.

### Validation of the risk model

With the help of ‘survival’, ‘survminer’, ‘heatmap’ as well as ‘glmnet’ package of R (version 4.1.1) statistics software, we calculated whole OC patients of subgroups including low- as well as high-risking parts. Kaplan–Meier curve as well as ROC (AUC) curve could be used to test the accuracy established model based on the R packages ‘survMiner’ and ‘survival’. Additionally, we tested whether the model would be a separate prognosis t indicating of OC sick persons by using univariate Cox regression (uni-Cox) as well as the multivariate Cox regression (multi-Cox). We conducted t-distributed stochastic neighbor embedding (t-SNE) and Princiapl Componrnts Analysis to visualize prognosis model.

### Nomogram

We included age, sex, stage, and TNM classification clinical characteristics in our analysis, constructed patient outcome nomograms for clinical utility, and compared predicted and actual probabilities using ‘rms’ packages in R. The concordance was assessed by plotting the calibration curves.

### The investigation of the tumor immune microenvironment

Using the CIBERSORT^28^ and ssGSEA algorithms^29^, we evaluated the infiltration status of immune cells. To further explore the TME of OC patients, we calculated the patients’ Stromal score, Immune score and ESTIMATE score using the ‘limma’, ‘estimate’ and ‘ggpubr’ packages in R. Additionally, we applied R package ‘maftools’ to assess as well as aggregate mutation information in OC patients. Data of the immune subtype was downloaded on TIMER (http://timer.comp-genomics.org/) .

### Exploration of the model in the clinical treatment

We applied R package "pRRophetic" (version 0.5) to assess the treatment responses, determined by the semi-maximum inhibited concentration (IC50) of Cancer Drug Sensitivity Genomics (GDSC) for every OC patient (https://www.cancerrxgene.org/). The data of drug sensitivity analysis are from the website (https://discover.nci.nih.gov/cellminer/home.do).

### Functional analysis

Using the ‘clusterProfiler, ‘enrichplot’ and ‘ggplot2’ package of R software to conduct GO enrichment analysis and KEGG enrichment analysis^30–32^. Further, we performed Gene Set Enriched Analysis (GSEA) (version 4.0.3) to explore the potential biological process and risk pathways between the high- and low-risk groups. The significant pathways and biological process were enriched with FDR < 0.05. Cytoscape (version 3.6.1) was applied to establish the network between lncRNAs and mRNAs for visualization.

### Statistical analysis

All data processing, statistical analysis, and plotting were conducted in the R (Version 4.1.3) platform. Wilcoxon rank-sum test was utilized for analyzing the difference between the two groups, while Pearson’s or Spearman’s correlation test was used for correlation. *P* < 0.05 was regarded as statistical significance. Among the statistical significance markers in all pictures, *, *P* < 0.05; **, *P* < 0.01; ***, *P* < 0.001; ns, no significance.

### Ethical approval

All analyses were based on previous published studies; thus no ethical approval and patient consent are required.

## Results

### Screening of fatty acid metabolism-related lncRNAs

**Figure 1:**
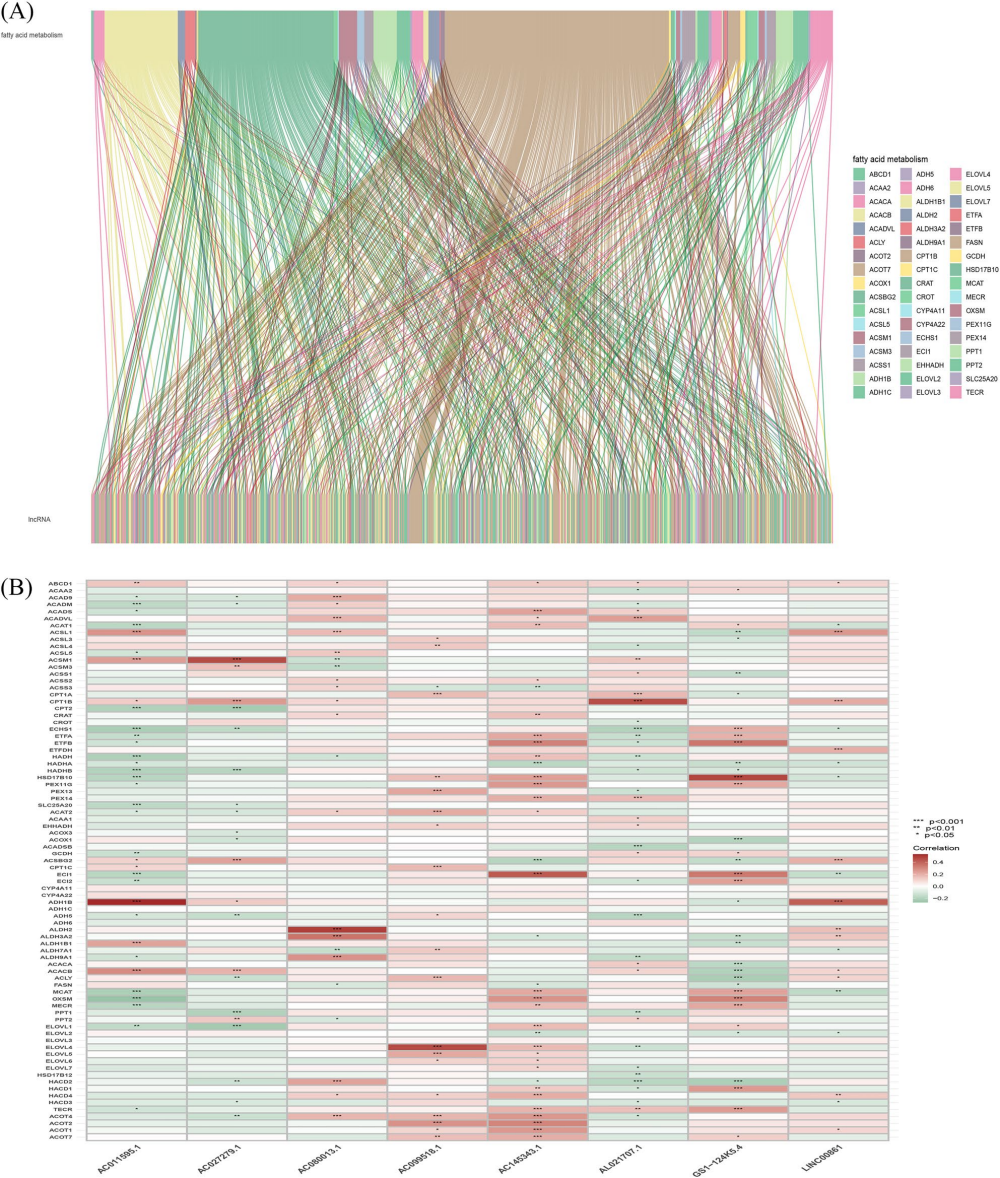
Identification of fatty acid metabolism-related lncRNAs in OC patients. (**A**) Sankey relation diagram for 92 fatty acid metabolism genes and fatty acid metabolism -related lncRNAs. (**B**) Heatmap for the correlations between fatty acid metabolism genes and the 8 prognostic fatty acid metabolism -related lncRNAs.

A total of 14,056 lncRNAs (Appendix D1) were extracted from TCGA database and 92 fatty acid metabolism-related genes were collected from Gene Cards (Appendix D2). Pearson correlation analysis identified 595 fatty acid metabolism-related lncRNAs. The correlation between fatty acid metabolism genes, such as MCAT and ADH6, and lncRNAs were shown in (Appendix D3). The fatty acid metabolisml-lncRNA coexpression network was visualized using the San-key diagram in (Fig. 1A) to show the correspondence between mRNA and lncRNA. The correlation between fatty acid metabolism genes and fatty acid metabolism-related lncRNAs was shown in (Fig. 1B).

### Construction of prognostic model

**Figure 2:**
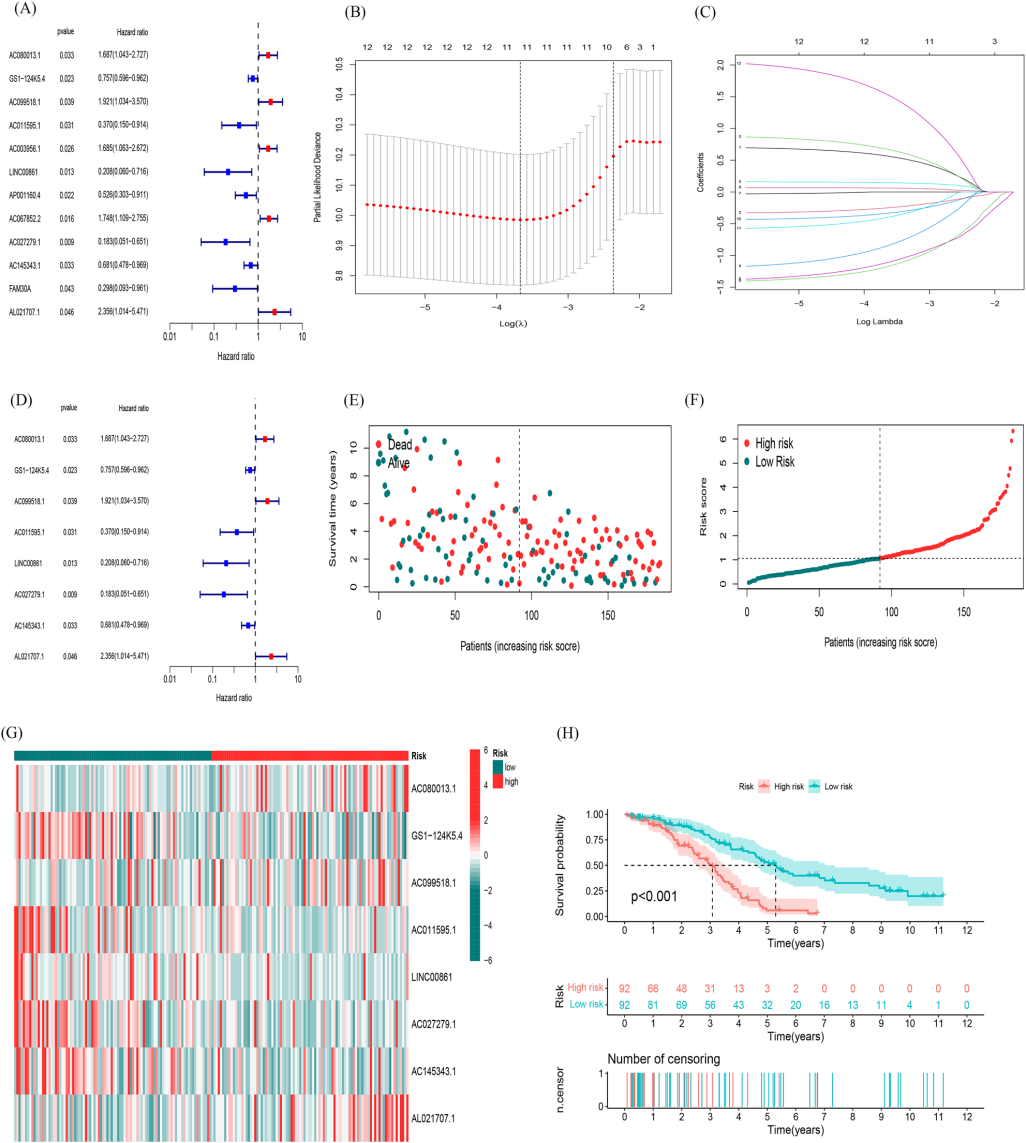
Construction and validation of prognostic model in the TCGA training set. (**A**) Univariate Cox regression analysis revealed that the 12 lncRNAs significantly correlated with OS. (**B**) The LASSO coefficient profile of fatty acid metabolism -related lncRNAs. (**C**) The tenfold cross-validation for variable selection in the LASSO model. (**D**) Multivariate Cox regression analysis showed 8 independent prognostic lncRNAs. (**E**) Different patterns of survival status and survival time between the high-risk and low-risk groups for the TCGA training set. (**F**) Distribution of fatty acid metabolism-related lncRNAs model-based risk score for the TCGA training set. (**G**) Clustering analysis heatmap shows the expression standards of the 8 prognostic lncRNAs for each patient in the TCGA training set. (**H**) Kaplan–Meier survival curves of the OS of high-risk and low-risk patients in the TCGA training set.

Univariate Cox regression analysis was applied to identified 12 fatty acid metabolism-related lncRNAs that had prognostic significance for OC in the training set (Fig. 2A). LASSO Cox regression analysis was performed for the further screening and identified 11 lncRNAs (Fig. 2BC). Multivariate Cox regression analysis was used to distinguish powerful prognostic lncRNAs. Eight fatty acid metabolism-related lncRNAs (Table 1) were used to construct a risk model to assess the prognostic risk of patients with OC (Fig. 2D).

**Table 1:** Eight fatty acid metabolism-related lncRNAs.

Id	HR	HR.95L	HR.95H	*p* value
AC080013.1	1.68684853	1.043458166	2.726949728	0.032879559
GS1-124K5.4	0.757028256	0.595723207	0.962010164	0.022801097
AC099518.1	1.92121658	1.033813846	3.570346017	0.038909743
AC011595.1	0.369732196	0.149557979	0.914039472	0.031193106
LINC00861	0.207728398	0.06027969	0.715847859	0.012791403
AC027279.1	0.183083788	0.051456366	0.65141937	0.008745965
AC145343.1	0.680723821	0.478203098	0.969012794	0.032787441
AL021707.1	2.355577286	1.014189288	5.47111315	0.0462906

We calculated risk score with the formula: risk score = AC080013.1 × (0.711824704621389) + GS1-124K5.4 × (− 0.361089508162097) + AC099518.1 × (0.856525829201051) + AC011595.1 × (− 1.38982864656339) + LINC00861 × (− 1.69408455780236) + AC027279.1 × (− 1.48090255405577) + AC145343.1 × (− 0.434071675192394) + AL021707.1 × (2.27209817622482).

According to median value of the prognostic risk grade, OC patients were categorized into low- and high-risk groups. The distribution of risk grades between the low-risk and high-risk groups is shown in (Fig. 2E). The survival satus and survival time of patients in the high-risk and low-risk groups are depicted in (Fig. 2F). This result demonstrates the good homogeneity of our included samples and the ability of the risk model to discriminate the included samples. The relative expression standards of the 8 fatty acid metabolism-related lncRNAs for every patient are shown in (Fig. 2G). According to the survival analysis, the OS of those in the low-risk group was longer than those in the high-risk group (*P* < 0.001, Fig. 2H), which indicated our risk model l can well predict the survival time of patients with different risk scores.

**Figure 3:**
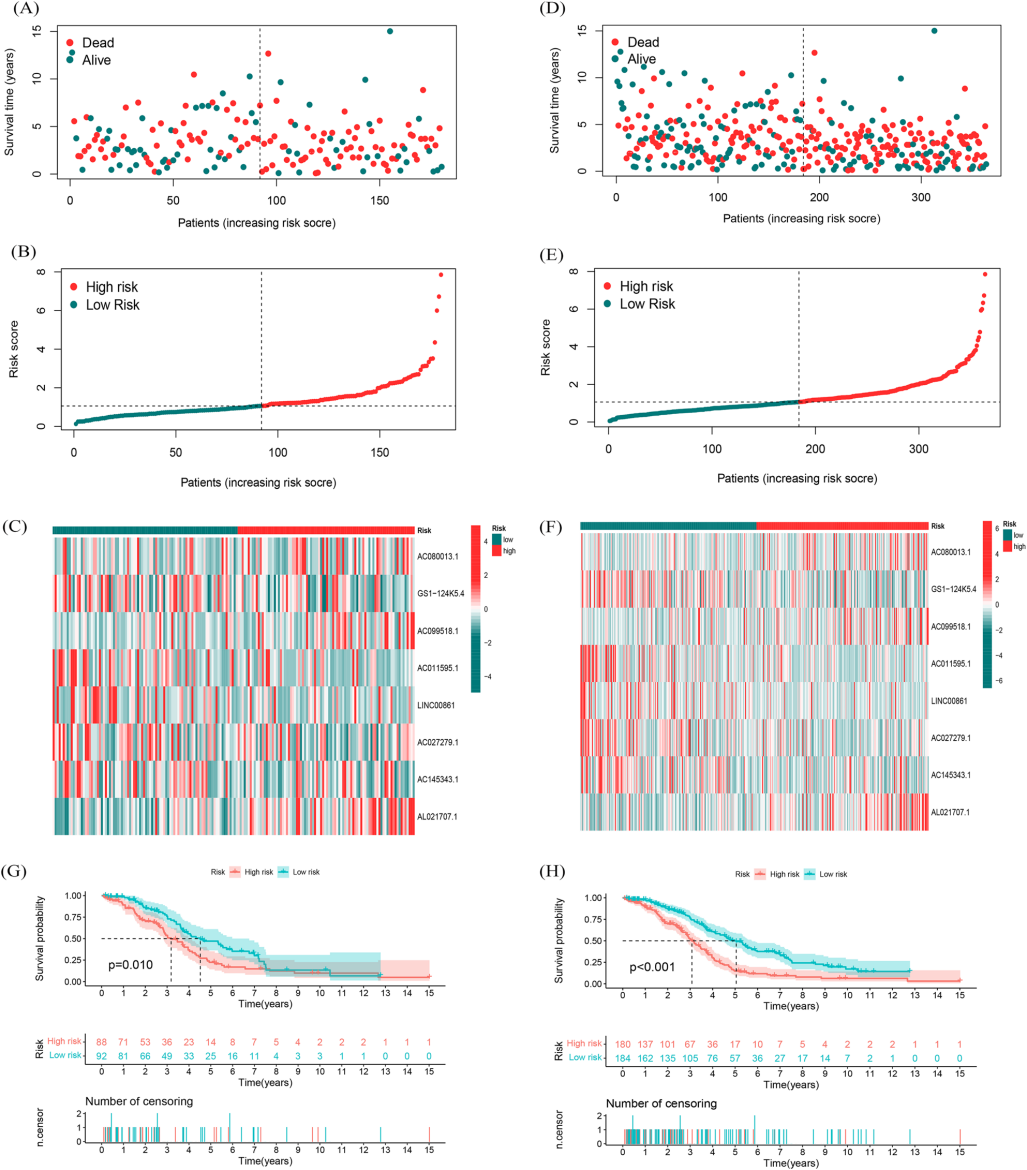
Prognostic value of the risk model of the 8 fatty acid metabolism -related lncRNAs in the TCGA testing and entire sets. (**A**) Distribution of fatty acid metabolism-related lncRNA model-based risk score for the testing set. (**B**) Patterns of the survival time and survival status between the high-risk and low-risk groups for the testing set. (**C**) Clustering analysis heatmap shows the display levels of the 8 prognostic lncRNAs for each patient in the testing set. (**D**) Distribution of fatty acid metabolism-related lncRNA model-based risk score for the entire set. (**E**) Patterns of the survival time and survival status between the high-risk and low-risk groups for the entire set. (**F**) Clustering analysis heatmap shows the display levels of the 8 prognostic lncRNAs for each patient in the entire set. (**G**) Kaplan–Meier survival curves of the OS of patients in the high-risk and low-risk groups in the testing set. (**H**) Kaplan–Meier survival curves of the OS of patients in the high-risk and low-risk groups in the entire set.

The uniform formula was used to calculate risk scores for each patient in the test and the entire sample in order to determine the prognostic capabilities of the established model. Figure 3 shows the distribution of risk grades, mode of living situation, survival time, and expression of the fatty acid metabolism-related lncRNAs in the testing set (Fig. 3A, B and C) as well as entire set (Fig. 3D, E and F). The samples in the two sets were evenly distributed and could be well divided into different risk subgroups. Among the Kaplan–Meier analysis conducted of the testing set and the entire set, there was no difference in the results: OS in OC patients with higher risk scores was worse than that of patients with lower risk scores (Fig. 3GH, P = 0.01 in testing set, *P* < 0.001 in entire set).

### Independent prognostic analysis

**Figure 4:**
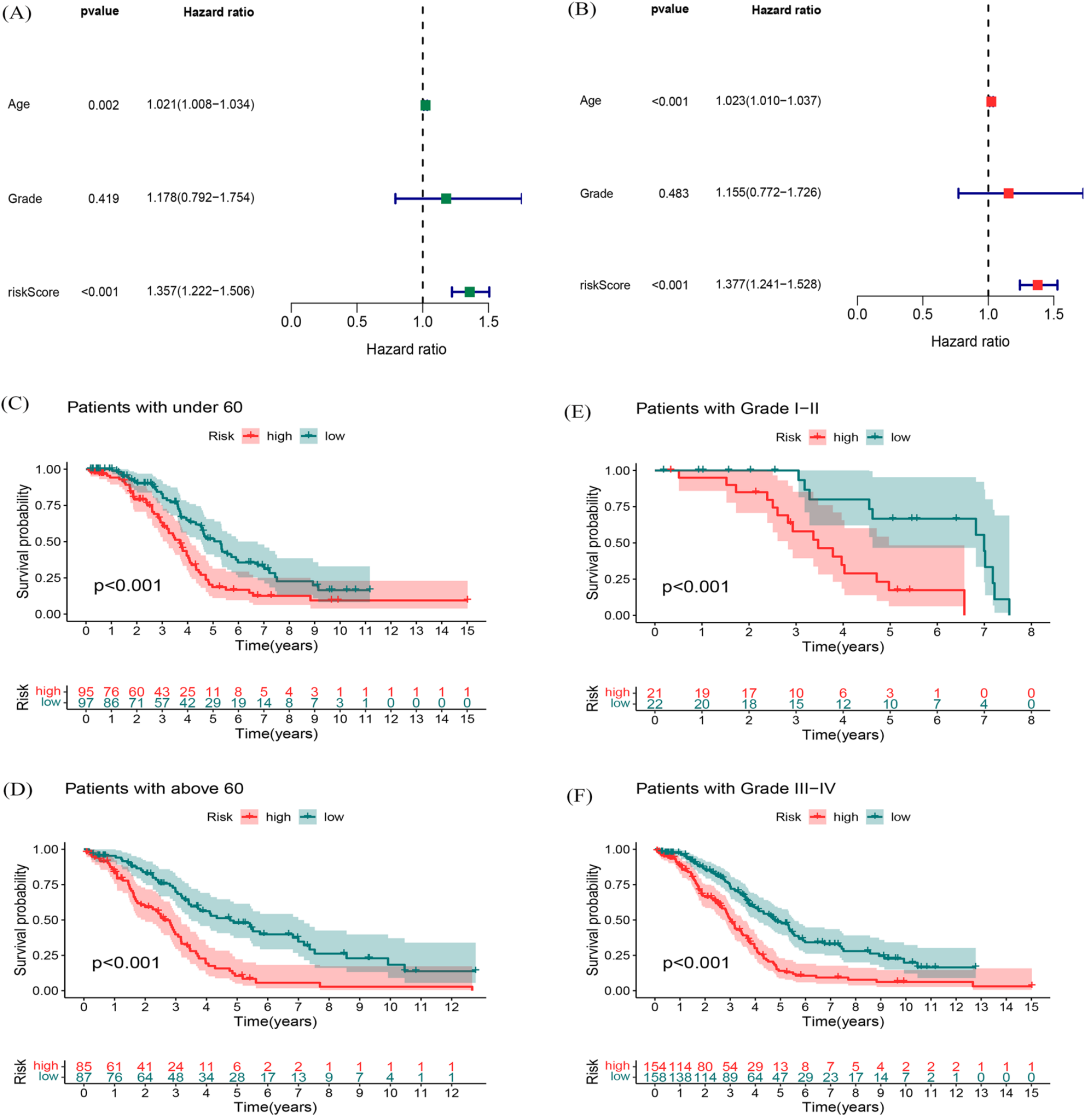
Association of the prognostic signature with clinical features. (**A**) forest plots of univariate Cox analysis. (**B**) forest plots of multivariate Cox analysis. (**C**–**D**) with age. (**E**–**F**) with pathological grade.

We focus on expression of associated fatty acid metabolism-related lncRNA prognosis characteristic with age as well as grade of pathological OC patients. The independent value of the prognostic signature was assessed by univariate and multivariate Cox regression analyses. Univariate regression analysis showed that age (HR: 1.023, 95% CI: 1.010–1.037, *P* < 0.001) and risk score (HR: 1.377, 95% CI: 1.241–1.528, *P* < 0.001) were associated with OS of OC patients (Fig. 4A). Multivariate Cox regression analysis showed that age (HR: 1.021, 95% CI: 1.008–1.034, *P* = 0.002) and risk score (HR: 1.357, 95% CI: 1.222–1.506, *P* < 0.001) were independent prognostic factors for OC patients (Fig. 4B). Furthermore, we performed stratification analysis to estimate if our model is applicable to OC patients with distinct age as well as grade of pathology. In the entire set, subjects got into two groups according to a cutoff value (age = 60), including an older group of OC patients (n = 281) and a younger group (n = 306). Furthermore, we calculated the risk score of two groups by fatty acid metabolism-related lncRNA signature. The OS between the high- and low-risk groups in the younger patient group (*P* < 0.001, Fig. 4C) and older patient group (*P* < 0.001, Fig. 4D) were significantly different. Similarly, entire set were also divided into Grade I–II (n = 75) and Grade III–IV (n = 496). The fatty acid metabolism-related lncRNA signature calssified two groups into the high- and low-risk group. Figure 4E, F indicated that the OS between the two groups were significantly different similarly, survival time of patients in the high-risk score group was significantly lower than that in the low-risk group. Above results suggested that the fatty acid metabolism-related lncRNA signature could independently predict overall survival of OC patients.

### Nomogram

**Figure 5:**
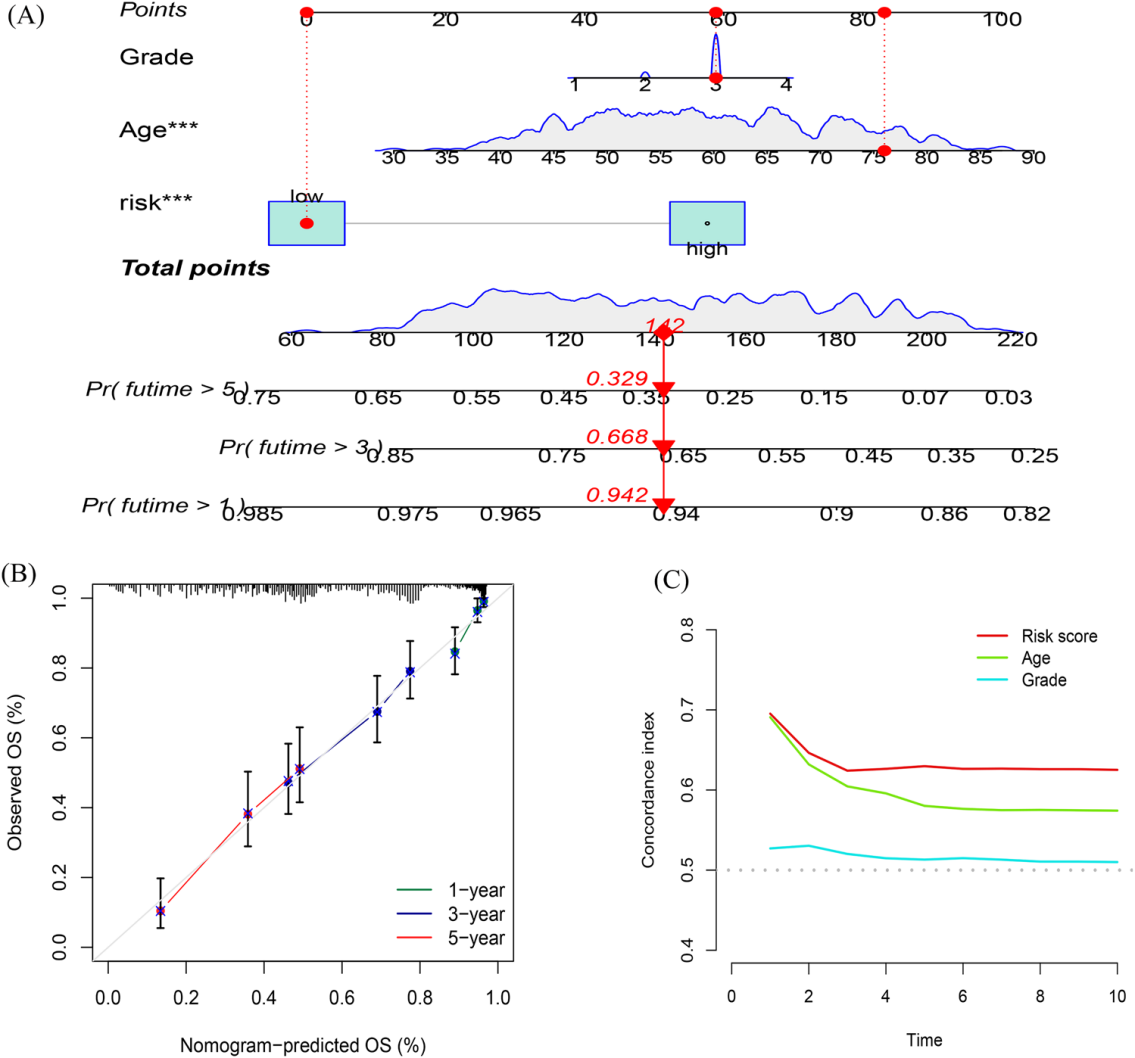
Construction and validation of the nomogram. (**A**) The nomogram predicts the probability of the 1-, 3-, and 5-year OS. (**B**) The calibration plot of the nomogram predicts the probability of the 1-, 3-, and 5-year OS. (**C**) Concordance indexes of the risk score and clinical characteristics.

In order to better forecast 1,3,5-y living proportion in OC patients, we established a nomograph which summed prognosis model as well as clinic information, containing age and pathological grade. As indicated in Fig. 5A, by scoring each item according to the actual situation, patients could obtain a total score that predicts their survival in the 1,3, 5-y range. Then verify the prediction accuracy of this nomogram. The blue line represents the observed survival rate, and the gray line represents the optimized survival rate, showing a good fit among observed and optimized values (Fig. 5B). Concordance index also showed the accuracy of risk model (Fig. 5C). Surprisingly, the risk score showed the highest index of concordance.

### Assessment of the risk model

**Figure 6:**
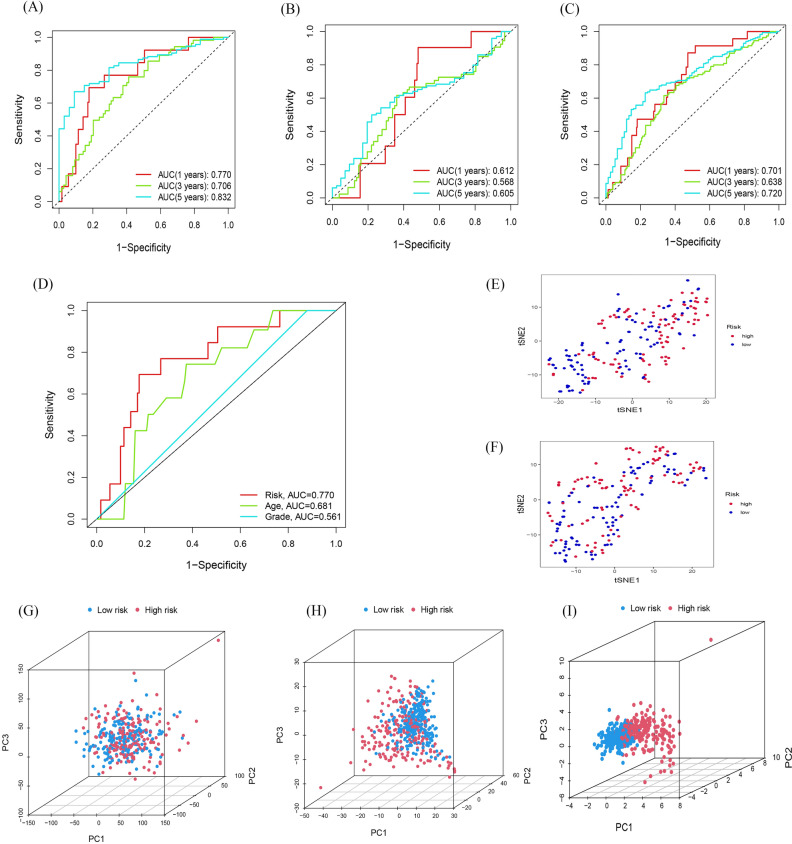
Assessment of the prognostic risk model. (**A**) The 1-, 3-, and 5-year ROC curves of the train set. (**B**) The 1-, 3-, and 5-year ROC curves of the test set. (**C**) The 1-, 3-, and 5-year ROC curves of the entire set. (**D**) ROC curves of the clinical characteristics and risk score. (**E**–**F**) t-SNE analysis based on training set and testing set. (**G–I**) PCA between the high-risk and low-risk groups based on entire gene expression profiles, 92 fatty acid metabolism genes and risk model based on the representation profiles of the 8 fatty acid metabolism-related lncRNAs.

Time dependent receiver operating characteristic (ROC) was used to assess the sensitivity and specificity of the model for prognosis. We also use the area under the ROC curve (AUC) to illustrate the ROC results. The AUC of train group at 1, 3 and 5 years was 0.770, 0.706 and 0.832, and that of testing set at 1, 3 and 5 years was 0.612, 0.568 and 0.605, and that of entire set was 0.701, 0.638 and 0.720, separately (Fig. 6A, B, and C). The AUC of the risk grade was also higher than the AUCs of other clinicopathological characteristics, showing that the prognostic risk model of the 8 fatty acid metabolism-related lncRNAs for OC was comparatively dependable (Fig. 6D, AUC of risk score = 0.77; of age = 0.681; of grade = 0.561). In order to further verifies the group ability of the fatty acid metabolism-related lncRNAs model, t-SNE analysis were conducted to test the difference between the high and low risk groups in the traing and testing set (Fig. 6EF). Furthermore, Principal-component analysis (PCA) analysis was also conducted to verify the difference between the high and low-risk based on the based on the whole genome expression set, 92 fatty acid metabolism-related genes and risk model classified by the expression profiles of the 8 fatty acid metabolism-related lncRNAs (Fig. 6G, H, and I). Results showed that the high-and low-risking parts had distinct distributions, suggesting that the prognostic signature can accurately differentiate between these groups.

### Identifiction of immune infiltration status in OC

**Figure 7:**
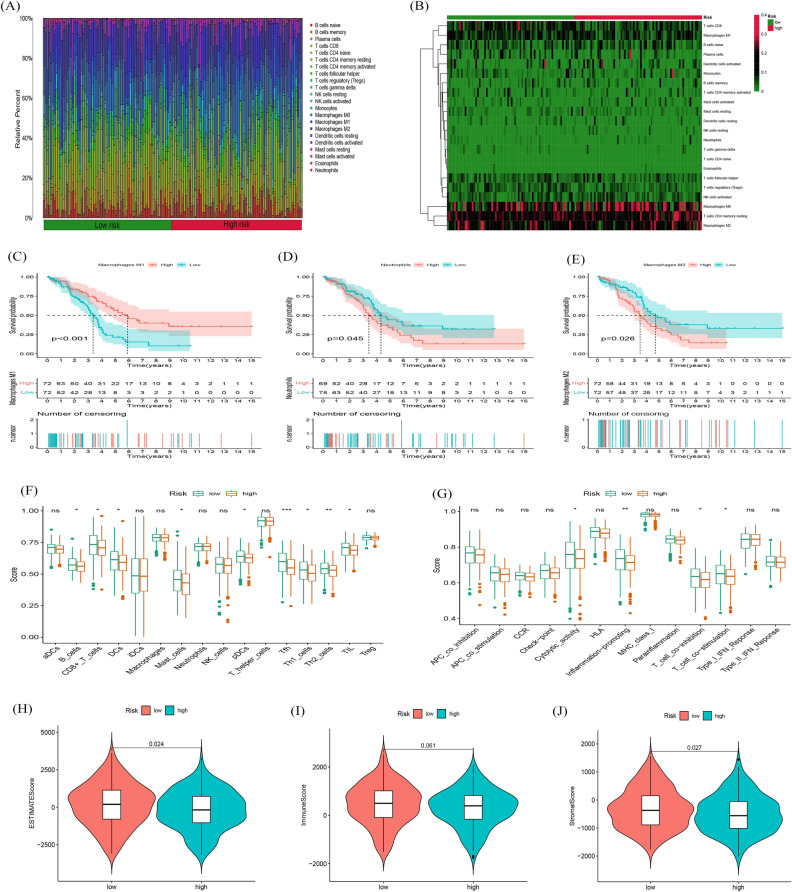
Stratification Analysis of the fatty acid metabolism-related lncRNA prognostic risk score in immune features. (**A**) Heatmap of 22 tumor-infiltrating immune cell types in low-risk and high-risk groups. (**B**) Bar chart of the proportions for 22 immune cell types. (**C**–**E**) K-M survival analys-is based on immune cells. (**F**) The score of immune functions comparing high-risk and low-risk groups by ssGSEA Score. (**G**) The score of immune cells comparing high-risk and low-risk groups by ssGSEA score. (**H**–**J**) The assessment of TME scores between high- and low-risk groups.

Here, we conducted the CIBERSORT algorithm to find out immune cell infiltration in OC patients. Figure 7A, B visualized the proportion of 22 immune cell components in high- and low-risk groups of OC patients. Further, K-M survival analysis showed that the immune cells, including Neutrophils, Macrophages M1 and M2 could better distinguished the OC patients between high- and low-risk groups (Fig. 7C, D and E, P < 0.001, *P* = 0.045, *P* = 0.026). Subsequently, the results of ssGSEA algorithm showed that infiltration of Tfh, Th2_cells, Th1_cells, pDCs, Mast_cells, DCs, B_cells were remarkably increased in the low-risk subgroup (Fig. 7F). The immune functions such as cytolytic activity, inflammation-promoting, T cell co-inhibition and stimulation were also higher in the low-risk group (Fig. 7G). As a result of all results, low-risking part appeared to have a higher immune permeation situation, which may contribute to antitumor effects. Furthermore, OC patients in the low-risk group also had significantly higher ESTIMATE, stromal, and immune scores, signifying a different TME from the high-risk group (Fig. 7H, I and J), these results above all indicate that compared with the low-risk group, patients in the high-risk group have more serious immune dysfunction and are in a more severe immunosuppressive state.

### Somatic mutation landscapes in OC

**Figure 8:**
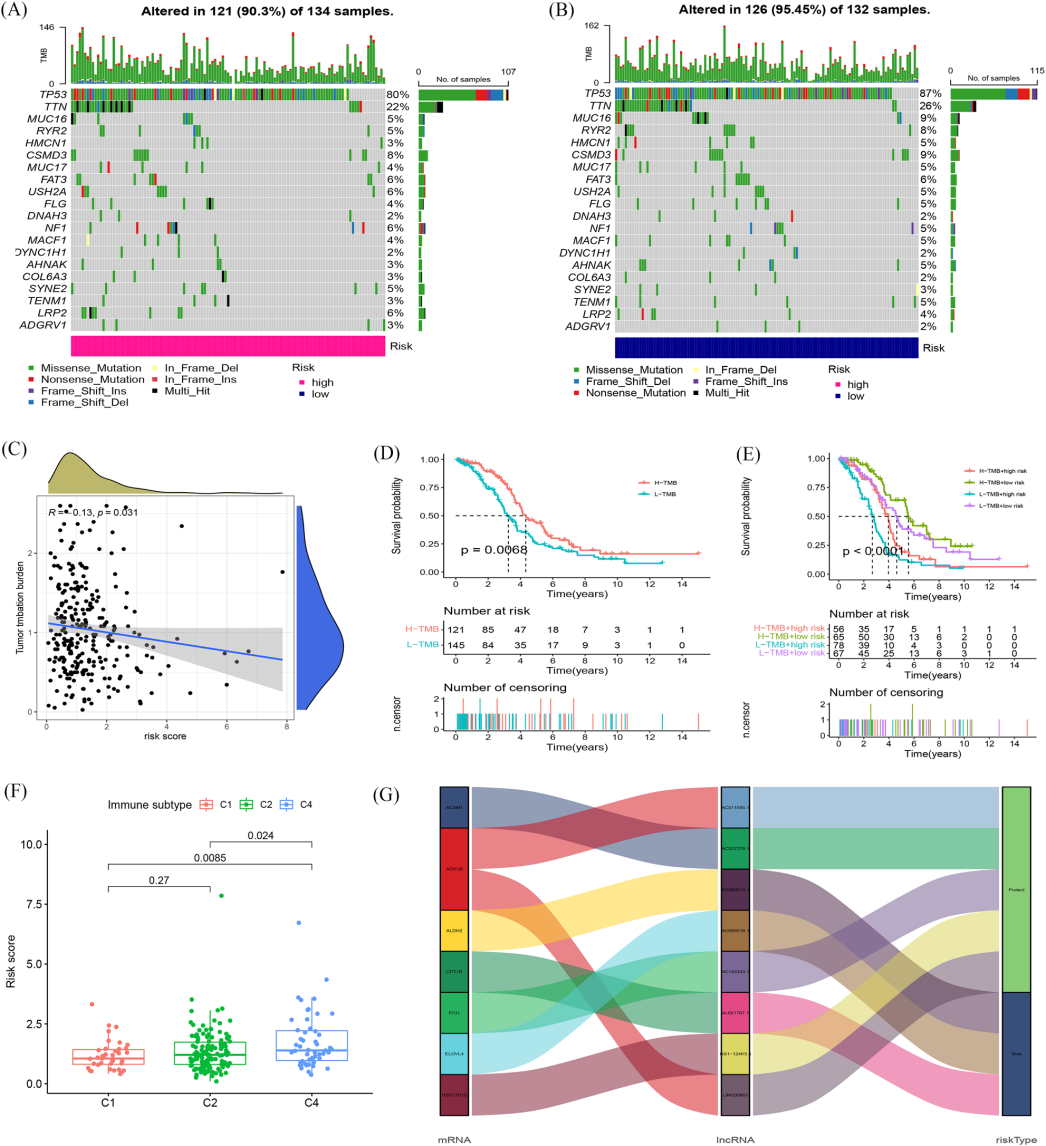
Exploration of Tumor mutation burden and visualization of lncRNAs networks. (**A**) Waterfall plot displays mutation information of the 20 genes with high mutation frequencies in the high-risk group. (**B**) Waterfall plot displays mutation information of the 20 genes with high mutation frequencies in the low-risk group. (**C**) The correlation between risk score and TMB. (**D**–**E**) Kaplan–Meier survival curves of the OS of patients in the high and low TMB and subgroups (**F**) The correlation between risk score and immune subtype. (**G**) Sankey diagram shows the connection degree between the fatty acid metabolism-related lncRNAs, fatty acid metabolism genes and risk types.

We studied the somatic mutation landscapes in ovarian cancer patients. By comparison, about 90.3% of the samples had gene mutations in high-risk samples, and 95.43% of the samples had mutations in low-risk samples. The top 20 driving genes with the highest change frequency in the high-risk and low-risk groups were shown in Fig. 8A, B. Subsequently, the correlation test was conducted between the fat metabolism-related lncRNA risk models (Fig. 8C, R = 0.13, *P* = 0.031), showing that the fatty acid metabolism-based classifier index had a weak correlation with TMB. Moreover, we tested whether the TMB related risk model was able to accurately predict the OS outcome, as shown in (Fig. 8D, E). The results show that the fatty acid metabolism-related lncRNAs model may have greater prognostic significance in OC patients.

Further, we tested whether a risk model utilizing fatty acid metabolism-related lncRNAs could distinguish between the different immune subtypes using the immune subtype data (Appendix D4) from TIMER2.0 (Fig. 8F). The results show that the risk model has a high ability to identify immune subtypes. Furthermore, fatty acid metabolism-related genes, 8 fatty acid metabolism-related lncRNAs, and risk types were included in the Sankey network (Fig. 8G). These results may provide some insights into the role of fatty acid metabolism-lncRNAs in OC oncogenesis, for example, AC011595.1, AC027279.1, GS1-124K5.4, AC080013.1, and LINC00861 may cooperate with each other to play a protective role in slowing down tumor progression in OC, while AC099518.1 and AL021707.1 act synergistically as tumor-promoting risk factors.

### Functional enrichment

**Figure 9:**
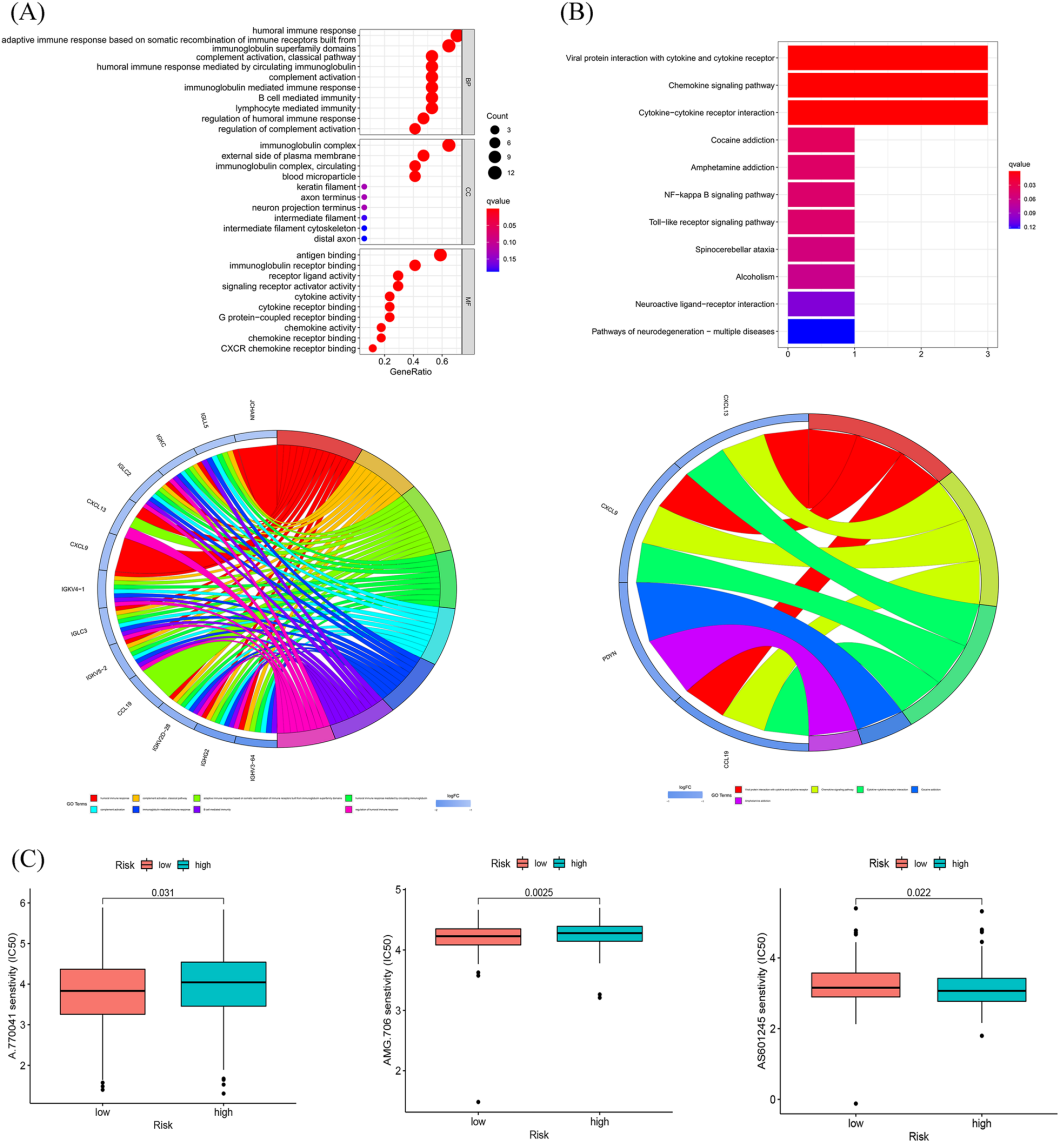

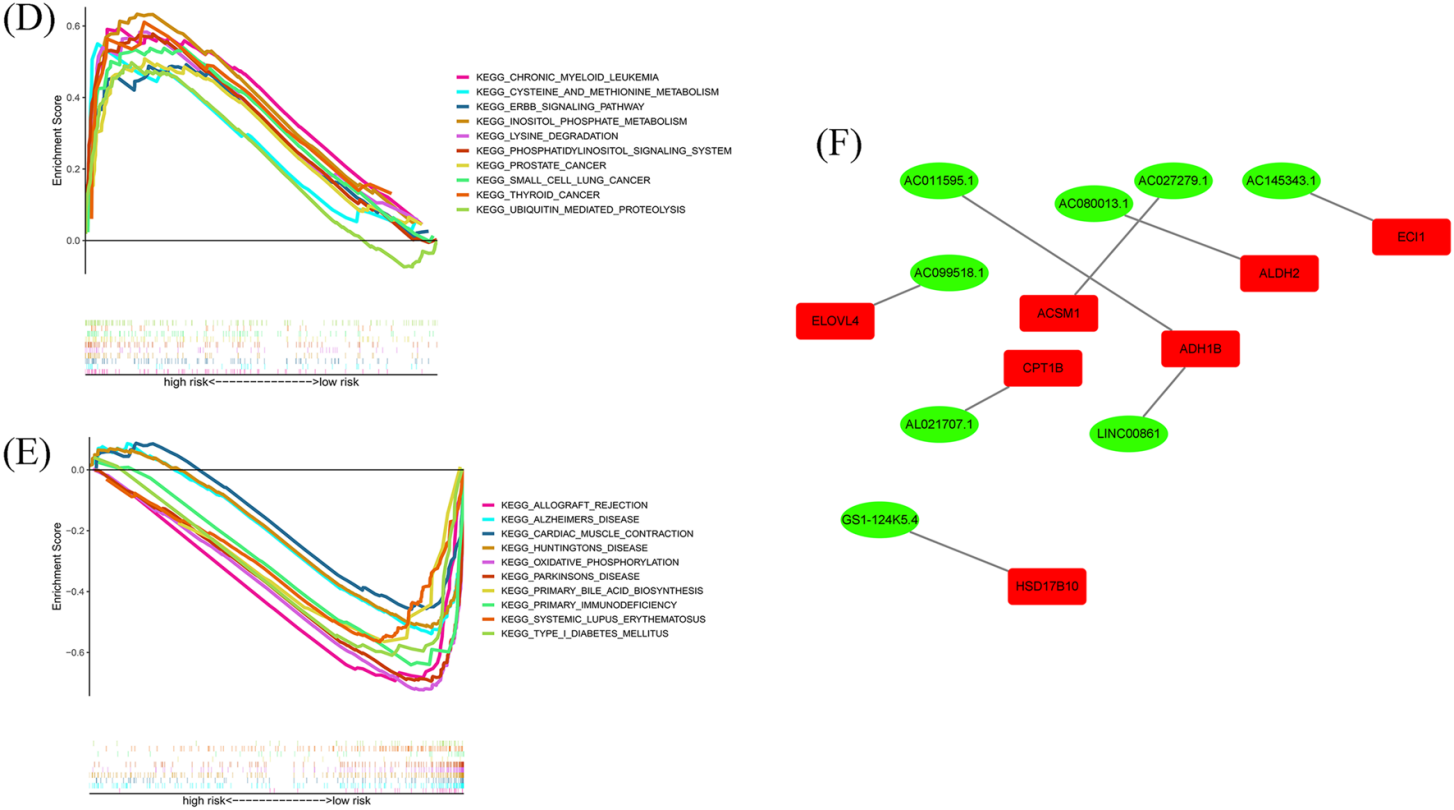
Functional analysis. (**A**) Top 10 classes of GO enrichment terms in biological process (BP), cellular component (CC), and molecular function (MF) based on 8 fatty acid metabolism-related lncRNAs. (**B**) Top 30 classes of KEGG enrichment terms. (**C**) Drug sensitivity analysis. (**D**) Gene set enrichment analysis of the top 10 pathways significantly enriched in the high-risk group. (**E**) Gene set enrichment analysis of the top 10 pathways significantly enriched in the low-risk group. (**F**) Construction of 8 fatty acid metabolism-related lncRNAs and metabolism genes networks.

Using enrichment analysis based on the 8 fatty acid metabolism lncRNA signature, we aimed to understand the biological processes that might be involved. As shown in Fig. 9A, GO enrichment analysis presented that the enrichment of multiple biological process (BP), cell component (CC), and molecular function (MF) are remarkably enriched in humoral immune response, complement activation classical pathway, immunoglobulin complex, immunoglobulin complex circulating, antigen binding and immunoglobulin receptor binding. KEGG study revealed that it mainly connected with viral protein interaction with cytokine and cytokine receptor, chemokine signaling pathway and cytokine − cytokine receptor interaction (Fig. 9B). Interestingly, to discern potential drugs targeting our lncRNA model for treating OC patients, we used the pRophetic algorithm based on the half-maximal inhibitory concentration (IC50) provided in the Genomics of Drug Sensitivity in Cancer (GDSC) database. The IC50 of A.770041 and AMG.706 were higher in the high-risk group (Fig. 9C), indicating that high-risk group patients were more sensitive to these drugs. Additionally, to better understand differences in biological functions, we utilized GSEA software to analyze the high-risk and low-risk groups in the KEGG pathway (Fig. 9DE). Pathways such as inositol phosphate metabolism and chronic myeloid leukemia were significantly enriched in the high-risk group, while pathways such as oxidative phosphorylation and parkinsons disease were significantly enriched in the low-risk group. Finally, we established an interation network using Cytoscape to visualize the co-expression between the lncRNAs and mRNAs (Fig. 9F), some of the key interacting mRNAs include ELOVL4, ACSM1, ADH1B, ALDH2, ECL1, and HSD17B10.

## Discussion

Ovarian cancer has the highest mortality rate of gynecological cancer, surpassing cervical cancer^33^. Heterogeneity and difficulty in early diagnosis of ovarian cancer also lead to the uncertainty of prognosis for OC patients, which brings significant challenges to the accurate treatment of OC^2^. At present, the internationally commonly used ovarian cancer malignancy scoring systems for the diagnosis of OC include the Malignancy Risk Index (RMI)^34^ and the Ovarian Cancer Risk Algorithm (ROCA)^35^. Still, these evaluation systems are suitable for a disease with many subtypes such as OC. In addition, these algorithms are mainly based on the clinical manifestations of OC and cannot accurately identify the molecular mechanism of the disease. Therefore, a newer, more applicable, and accurate OC risk scoring system is needed. It is worth mentioning that, with the gradual popularization and in-depth application of model algorithms in disease prediction, more and more traditional disease scoring standards and models have been improved^36,37^. These algorithms often use more accurate and quantifiable indicators (such as blood biochemical tests^38^, quantifiable imaging results^39^, etc.), and can incorporate more personal and personalized data types into the diagnosis, treatment and prognosis of patients. This can make the clinical treatment management of the disease more accurate and facilitate the formulation of better treatment plans.

Nowadays, with the deepening of the research on the tumor process, tumor cell metabolic reprogramming was considered a crucial part of tumor process regulation^40,41^. Interestingly, recent extensive anthropological studies have shown that high absorbance of lipids is related to an increasing incidence in OC^42,43^; this also reflects from the side that lipid metabolism may be closely related to the progression of OC. The overall lipid metabolism process includes fatty acid oxidation and synthesis. Previous studies have demonstrated that fatty acid synthase (FASN or FAS) and the de novo synthesis of fatty acids are hyperactivated in OC^44^. The up-regulated levels of FASN or FAS are thought to be closely associated with poor prognosis and clinical grading of OC patients^44,45^, and also mediates the resistance of OC cells to the first-line therapy cisplatin^46^. In addition to the changes to the tumor cells themselves, changes in the lipid metabolism level of adipocytes in the tumor microenvironment can also cause dramatic changes in the activity of immune infiltrating cells in the TME and tumor cells themselves, ultimately leading to changes in tumor progression^47–49^. In addition, many recent studies have confirmed that lncRNA, a post-transcriptional modifier, can be cross-linked with the reprogramming of fatty acid metabolism in tumor cells^50–52^. Therefore, based on the above evidence, we established a novel OC predictive risk model from fatty acid metabolism-related lncRNAs and explored how these lncRNAs affect OC progression.

In our study, eight key fatty acid metabolism-related lncRNAs were screened. Except for AL021707.1, AC145343.1, and LINC00861, other lncRNAs are rarely studied. In the study by Miaolong Lu et al., AL021707.1 was considered involved in the N6-methyladenosin process and a potential therapeutic target for bladder cancer^53^.AC145343.1 was deemed associated with genomic instability mutations in liver cancer in the studies of Jianhua Wu and Dan-Ping Huang, and both studies considered AC145343.1 a risk factor for liver cancer progression^54,55^. Paradoxically, combined with the poor prognosis of the high-risk group, we also believe this lncRNA as a risk factor for OC. It is worth noting that the above two lncRNAs have not been studied in OC. For LINC00861, there are more related studies. The study by Hui Liu et al. suggested that LINC00861 is down-regulated in OC tissues, resulting in decreased activity of the PTEN/AKT/mTOR pathway, which leads to the progression of OC and the poor prognosis of patients with advanced OC^56^. In addition, a recent blockbuster study on eczema identified TRIB1/LINC00861 as one of the crucial variants, and this change is closely related to immune cell function^57^.

Looking back on our analysis of the immune characteristics of OC, it is not difficult to find that the infiltration degree of various tumor- permeating immune cells as well as activity of different immune responses have changed, and there are significant differences between high and low-risk groups. For example, compared with the low-risk group, the infiltration of Tfh (Follicular B helper T cells) in the samples of the high-risk group was significantly decreased. The cells can be generated during the differentiation of Th cells to Th1 or Th2 cells^58^ and participate in the production and maintenance of B cell germinal centers by secreting cytokines, which play an essential role in the humoral immune process of organisms^59,60^. This is consistent with our results in the GO enrichment analysis that humoral immunity is enriched at the very top. This has been shown to be a key regulator of tumor immune responses in various tumors^61,62^. The latest findings point out that the transfer of Tfh cells can inhibit the growth of OC cells^63^, while previous studies by Li Li et al. observed that Tfh can reduce the activation of co-cultured CD8 + T cells by affecting IL-10, thereby making the body decreased tumor clearance^64^. At the same time, we also noticed that there are significant differences in immune pathways such as cytolytic activity, pro-inflammatory process, among patients in different risk groups. Inflammation has always been an important link that cannot be ignored in OC, and it is often related to angiogenesis and apoptosis, reversal of chemotherapy resistance, improvement of systemic symptoms and prognosis, and many other aspects^65–67^. In OC, pro-inflammatory mediators that have been focused on include the IL-6 family, metallomatrix enzymes, and more. Among them, a large number of studies have shown that IL-6 can activate the JAK/STAT pathway, induce tumor proliferation, and can also intensify endothelial-mesenchymal transition and other cancer-promoting processes. Interestingly, we found that patients in the low-risk group had relatively higher pro-inflammatory activity scores, which may be due to the overall suppressed immune system in the high-risk group^68,69^. At the same time, we noticed that Francesca Bellora et al.’s 2014 study pointed out that tumor-associated macrophages in ovarian cancer patients can effectively trigger the cytolytic activity of NK cells through the release of IL-12/18 from the M1 phenotype^70^. It may be able to explain the difference in cytolytic activity among patients in different risk groups. In addition, there was no significant difference in the degree of NK cell infiltration between the high-risk group and the low-risk group in our immune cell infiltration analysis, which may imply the low functional state of NK cells in the high-risk group. From this point of view, it may be a potential OC immunotherapy target.

Admittedly, this study still has the following shortcomings. First of all, this research is according to analysis in bioinformatics technology, and results obtained need to be supplemented with corresponding animal or cell experiments to verify. In addition, the data in this study came from sample information in public databases, which may lead to bias in the analysis results.

This is the first comprehensive study of fatty acid metabolism-related lncRNAs in OC patients. The expression profiles of lncRNAs and heavy acid metabolism genes were programmed, and a risk score model and a nomogram were built based on eight fatty acid metabolism-related lncRNAs. This risk model has been proved to have good reliability and validity of OC prognosis prediction and can be used as a signature to describe the immune characteristics of OC. The above research aims to offer novel ideas and viewpoints of precise therapy of OC, at the same time, it also provides a better reference for more accurate clinical diagnosis and prognosis of OC.

### Supplementary Information

Supplementary Information.

## Data Availability

The datasets used or analyzed during the current study are available from the corresponding author on reasonable request.
